# Updates on Laboratory Evaluation of Feline Cardiac Diseases

**DOI:** 10.3390/vetsci8030041

**Published:** 2021-03-03

**Authors:** Alessandra Gavazza, Andrea Marchegiani, Lorenza Guerriero, Vanessa Turinelli, Andrea Spaterna, Sara Mangiaterra, Livio Galosi, Giacomo Rossi, Matteo Cerquetella

**Affiliations:** 1 School of Biosciences and Veterinary Medicine, University of Camerino, Via Circonvallazione 93/95, 62024 Matelica, Italy; andrea.marchegiani@unicam.it (A.M.); andrea.spaterna@unicam.it (A.S.); sara.mangiaterra@unicam.it (S.M.); livio.galosi@unicam.it (L.G.); giacomo.rossi@unicam.it (G.R.); matteo.cerquetella@unicam.it (M.C.); 2 Policlinico Veterinario Roma Sud, Via Pilade Mazza 24, 00173 Roma, Italy; lorenza.guerriero@libero.it; 3 IDEXX Laboratories-Italia S.r.l. Via G. Silva 35, 20149 Milano, Italy; Vanessa-Turinelli@idexx.com

**Keywords:** laboratory test, biomarker, cardiac disease, review

## Abstract

Laboratory tests can be altered in cardiovascular diseases and the investigation of specific tests or biomarkers may provide additional information about myocardial damage. Traditional laboratory tests, such as cell blood count, serum biochemistry, and coagulation, can be useful in investigating patients, but are not specific. However, markers like Troponin and Natriuretic Peptides may possibly furnish further data on myocardium damage and can be used in both studying and monitoring cats with cardiac disease. Moreover, the evaluation of the thyroid profile is very important as hyperthyroid cats concomitant cardiovascular diseases are very common and they can also be a direct consequence of endocrinopathy. The purpose of this manuscript is to provide the widest possible overview of what is present in the literature about the feline clinical pathology of heart diseases through a rational division of the main alterations of traditional tests and biomarkers.

## 1. Introduction

Laboratory tests, including biochemical, hematological and coagulative profile, as well as urinalysis, could be altered in cardiac diseases, but are not specific and not always correlated with the severity of the condition. As a consequence, the search into new tests or biomarkers able to furnish information on myocardial damage and that can be used to investigate and monitor patients with cardiac diseases in both cats and dogs is highly relevant [1]. Results of laboratory tests should be evaluated in the context of the medical history, physical examination, and other specific diagnostic evaluations, such as thyroid functionality, aiming at using a composite approach while assessing the condition.

## 2. Laboratory Tests

### 2.1. Biochemical Profile

Creatine kinase (CK) is a cytosolic enzyme present in skeletal muscle, cardiac muscle and brain. CK-MB is the isoenzyme present in cardiac muscle with relatively low activity in other tissues, while CK-MM is present in both skeletal and cardiac muscle. The elevation of total CK suggests muscular damage, and although the serum half-life in dogs is known and is less than 2 h, half-life in cats has never been published [2,3,4].

A study including more than 600 cats showed that increased serum CK can be found in 7.6% of different cardiac diseases (e.g., cardiomyopathy, congestive heart failure associated to degenerative valve disease, myocarditis and congenital cardiac defects) and in 6.7% of patients affected by cardiomyopathies (hypertrophic, restrictive, dilated and hypertrophic-obstructive). Arterial thromboembolism (ATE) resulted in the presence of 25% of patients diagnosed with cardiomyopathy and is possibly associated with an increase in serum CK activity [4]. Elevations in CK and Aspartate aminotransferase (AST) were also frequent in cats with acute or subacute myocardial necrosis [5], and markedly raised levels of CK (with concurrent thrombocytopenia) were evidenced in the case of a nine-year-old cat presenting with heart failure (HF) [6]. In another cat with endocarditis (associated to *Bartonella henselae*), albumin was mildly decreased and CK activity was increased [7]. Finally, the most common serum chemistry findings in a case series of 13 cats with infective endocarditis were: increased blood urea nitrogen, hypoalbuminemia, and hyperbilirubinemia [8]. 

Aspartate aminotransferase is a cytosolic and mitochondrial enzyme, and its activity is not tissue-specific, but its major sources are muscle and liver; plasma AST half-life is about 1.5 h [2,3]. 

Lactate dehydrogenase (LDH) is a cytosolic enzyme present in all cells, but only liver, muscle, and erythrocytes injuries may be accountable for an increase in its serum activity. LDH consists of five isoenzymes and LDH1 is the most represented in heart and kidneys, but total LDH is usually dosed. LDH half-life is lower than 6 h [2,3]. Hyperglycemia, azotemia, hypercholesterolemia, and hypocalcemia are other laboratory findings that, together with elevations in muscle markers alanine aminotransferase (ALT), AST, and CK, may be associated to ATE. The reason for these alterations is not always clear, but in cats, hypocalcemia was associated with marked hyperphosphatemia and hyperkalemia and may result in being second to hyperphosphatemia as intracellular phosphorus is released and complexes with calcium. Azotemia likely further suggest decreased systemic perfusion. All cats with ATE had high AST and CK, possibly originating from ischemic muscle [9,10]. Concerning monovalent electrolytes and minerals, the most important alterations related to cardiac diseases include hyponatremia, seldom associated to congestive heart failure when the retention of water is greater than sodium, hyperkalemia and hyperphosphatemia secondary to the shift from intracellular to extracellular compartment during myopathies. Electrolyte disturbances are likely associated with reperfusion injury and the effects of decreased perfusion on renal function [10,11,12]. 

Finally, serum amyloid A (SAA) is one of acute phase proteins (APPs) in cats and is the most quickly responsive one to inflammatory triggers. In cats with generalized hypertrophy, SAA was not significantly increased in the overall population, but was higher compared to cats with focal or multifocal hypertrophy. It is uncertain whether this relationship is only present in cats with asymptomatic hypertrophic cardiomyopathy (HCM), or if it is related to the pathophysiology of the illness. Inflammation has been associated with congestive heart failure (CHF) in diverse species and cardiac diseases [13]. 

#### 2.1.1. Cardiorenal Syndrome

The term “Cardiorenal Syndrome” (CRS) refers to a “disorders of the heart and kidneys whereby acute or chronic dysfunction in one organ may induce acute or chronic dysfunction of the other” [14,15]. In 2015, the CRS Consensus Group chose to refer in veterinary patients to “cardiovascular-renal disorders (CvRD)”, including this way also the vascular system as well as the heart. The concept of CvRD emphasizes the inter-related functions of the two organs and suggests a complete and early investigation of heart function in nephropathic patients and of renal function in patients with cardiac disease. Unfortunately, the prevalence of CvRD in cats and dogs is unknown [16]. Azotemia is the most likely to be an altered variable in CvRD. It corresponds to an increase of hematic non-protein nitrogenous compounds: Urea (Ur) and Creatinine (Cr). Azotemia could be derived from decreased cardiac output and reduced urinary excretion of Ur and Cr. Urea is a protein metabolism product and is excreted by the renal system; consequently, its increased concentration is the result of a diminished glomerular filtration rate (GFR) that may occur in cases of shock, dehydration, and cardiovascular disease. Cr is filtered by the glomerulus and there is no tubular reabsorption. Therefore, it is a more accurate measurement of GFR than Ur; obviously, a reduced GFR affects both Cr and Ur [17]. The measurement of Ur, Cr and symmetric dimethylarginine (SDMA), together with urinalysis are essential for the diagnosis of CvRD. SDMA is a sensitive and specific renal biomarker and levels in serum increase as kidney function and GFR decrease [16,18]. The serum SDMA level was shown to be strongly correlated to GFR in cats regardless of whether these cats were either presenting azotemia or not [19]. In a retrospective longitudinal study involving 21 cats, SDMA levels increased up to 17 months earlier if compared to Cr, showing a much higher sensitivity (100% vs. 17%), but also lower specificity and predictive value [20]. Interestingly, in cats with hypertrophic cardiomyopathy (HCM) and diabetes mellitus (DM), independently of chronic kidney disease (CKD), only subjects with DM presented lower SDMA concentrations than controls, possibly for the effect of hyperfiltration. Additionally, serum SDMA concentrations in cats with HCM were not significantly different from healthy controls [21]. A recent study identifies a cardiorenal profile that included NT-proBNP, SDMA, Cr and 7 APPs. The APPs studied provide valuable information for cats with CHF and they may be helpful for understanding disease pathogenesis and for establishing prognosis in combination with other assessments. All cardiorenal biomarkers were positively correlated and higher in CHF cats, and high NT-proBNP and SDMA were associated with poor clinical outcomes [22].

Cystatin C (CysC) is a novel endogenous marker of GFR. It is a proteinase inhibitor, produced by nucleated cells, and numerous studies in dogs and humans confirm the superiority of serum CysC compared to Cr for the detection of renal damage. In healthy cats and cats with CKD, an excellent correlation with GFR was established for serum CysC compared to Cr [23,24]. 

#### 2.1.2. Cardiohepatic Syndrome

In humans, heart failure and liver disease often coexist because of complex cardiohepatic interactions, also present during systemic illnesses and diseases that affect both organs (infections, medicines, autoimmunity, inflammation, etc.). HF may lead to liver disease and liver disease could cause cardiac dysfunction and failure; this interaction is defined as “cardiohepatic syndrome” [25,26]. In a large feline retrospective study involving 260 cats with HCM, a slight increase in liver enzymes (ALT or AST) was found in about 70% of animals [27]. Another retrospective study on pericardial effusion in 146 cats with cardiac disease showed that CHF was the most common cause and that biochemical alterations were uncommon, except for AST activity, which often resulted in increases (85%). While AST activity may increase in cats with hepatic disease and/or myopathy, it can also increase in various other diseases such as anorexia, hyperthyroidism, and ATE. In this study, the overlap in AST activity between cats with and without cardiac disease made it difficult to assess whether slight to moderate increases in AST activity may also be a marker for cardiomyopathy in the cat [28]. Another recent study in cats also showed a significant increase in AST and alkaline phosphatase (ALP) with pleural effusion given by heart disease, compared to cats with pleural effusion given by other causes [29]. Nevertheless, no study has yet supported the presence of cardio-hepatic syndrome in cats, but based on such data, we may begin to speculate on it.

#### 2.1.3. Oxidative Stress

Oxidative stress is the imbalance between the production and neutralization of reactive oxygen species (ROS). Uncontrolled ROS overproduction leads to protein and lipid peroxidation, and damages the DNA strands, damaging and leading to cellular death. The main elements of the enzymatic antioxidant protection system are superoxide dismutase (SOD), converting the superoxide anion (O_2_^−^) into hydrogen peroxide, which is ultimately detoxified by catalase (CAT) and glutathione peroxidase (GPx). A recent study shows that the activities of SOD and CAT are different in cats with HCM, but the activity of CAT was only lower in asymptomatic subjects. This opens new perspectives for the administration of antioxidants to possibly stop the progression of the disease [30].

### 2.2. Hematological Profile

In human, the presence at the same time as HF, renal insufficiency (RI), and anemia determines a clinical triangle termed *cardiorenal anemia* [31]. The pathophysiology of anemia associated to HF is multifactorial and includes renal dysfunction and impaired/downregulated erythropoietin production [31,32], overproduction of proinflammatory cytokines such as tumor necrosis factor and interleukins [33], and expansion in plasma volume [34]. A preliminary study investigated the association between anemia and cardiovascular changes in cats caused by neuroendocrine activation, leading to circulatory volume overload and increased left atrial and left ventricular diastolic dimensions. This study revealed, in a small number of cats, that severely anemic subjects are more likely to have increased left heart dimensions than mildly anemic cats [35]. Conversely, an old case report described a Siamese in a “true polycythemia” secondary to hypoxia, which was associated to tetralogy of Fallot; the resultant decrease in the arterial partial pressure of oxygen stimulated an increased production of erythropoietin, thus causing the polycythemia [36]. 

Red blood cell distribution width (RDW) is a determination of anisocytosis, and in humans, it is considered to be a largely accessible marker, capable of predicting adverse outcomes in heart failure [37,38]. Similarly, RDW was significantly increased in cats with HCM and CHF compared to cats with HCM, but without CHF, and controls. A higher RDW is an autonomous predictor of cardiac death in cats with HCM without concurrent non-cardiac related illness [39]. Unfortunately, RDW is not likely to be a suitable prognostic indicator in cats due to differences in RBC physiology compared to human RBC. Feline RBCs have a shorter life span (70–80 days vs. 120 days human) and contain a greater number of hemoglobin sulfhydryl groups and limited glutathione stores. Thus, it is more predisposed to oxidative stress and possibly leads to a greater variability in normal RDW values with respect to humans [40]. 

Thrombocytopenia and leukocytosis were described as being present in approximately 90% of patients in several clinical cases of dogs with endocarditis but rare studies are present in feline medicine [41]. Moreover, hematological abnormalities, including leukocytosis characterized by neutrophilia and a regenerative left shift, associated with mild toxicity, were described in a clinical case of *Bartonella henselae* endocarditis in a cat [7]. Finally, a further case series of 13 cats with infective endocarditis showed inflammatory neutrophilia in seven patients and anemia in six [8]. 

### 2.3. Coagulative Profile

To examine the prevalence of thrombocytopenia and associated diseases in a feline population, an interesting study, but with a small case load, was performed in the early 1990s. Three cats with cardiac diseases also presented hemostatic disorders, including aortic or pulmonary thromboembolism, hemothorax, and disseminated intravascular coagulation [42]. A recent retrospective study performed at a UK hospital aiming to assess the prevalence of thrombocytopenia in 194 cats showed in subjects with cardiovascular disease a prevalence of 5% vs. 4% of cats with normal platelet count (P not significant) [43]. Conversely, a study on thrombocytosis observed this finding in 5 out of 51 cases of cardiovascular diseases: HCM, ATE, hypertension and thromboembolic disease. With regard to causes, iron deficiency is a commonly reported cause of reactive thrombocytosis in both humans and dogs and increased levels of thrombopoietic cytokines may be involved [44].

A frequent coagulative disease in cats is thromboembolic disease that involves either a locally formed (in situ) aggregation of platelets and other blood elements (thrombus) or other aggregates (embolus) that escape from its origin site and is carried by blood flow. Damaged endothelial cells promote thrombus formation [9]. Vasculitis or endocarditis can alter endothelial cells, or lead to exposure of subendothelium, each of which can lead to a prothrombotic state and platelet adhesion, aggregation, and secretion [45]. The high prevalence of ATE in cats with myocardial disease, particularly when associated with left atrial enlargement, suggests the presence of a hypercoagulable state before the beginning of thrombus formation [46]. The clinical incidence of thrombosis in cats has been connected with increased platelet hypersensitivity, decreased protein C and antithrombin activities, and increases in fibrinogen and factor VIII activity [9,46,47,48]. In cats with clinical signs of cardiomyopathy platelets results in hyperaggregable to adenosine diphosphate in vitro and there is no apparent correlation between the platelet count and the degree of the threshold aggregation response [48]. ATE has been reported as a sequela of feline HCM in 13%–17% of clinical cases and 41% of cases in a postmortem examination survey. ATE also occurred as a complication of dilated cardiomyopathy (DCM), unclassified cardiomyopathy (UCM), other forms of cardiac disease, neoplasia and hyperthyroidism [49,50]. In a study of 90 cats with ATE, primary underlying diseases were hyperthyroidism, cardiomyopathy (dilated, unclassified, hypertrophic obstructive, and hypertrophic), neoplasia, other [10]. 

There was no significant difference in Closure Time among healthy cats and cats with HCM, indeed it results slightly, but not significantly, extended in HCM cats. There were also no significant differences in cats between mild, moderate, or severe HCM [51]. 

The study of Bedard et al. in cats with HCM suggested that plasma thrombin-antithrombin complex appeared to be the most sensitive marker, followed by D-dimer and fibrinogen/fibrin degradation product (FDP). Using these markers, suspected evidence of activated coagulation and hypercoagulability was not strongly demonstrated in 45% of cats diagnosed with HCM. No differences in prothrombin time (PT) and activated partial thromboplastin time (aPTT) between cats with HCM and controls were found [52]. HCM and cardiomyopathy are associated in human patients to platelet activation that may be due to several factors, including increase P-selectin expression [53,54,55,56]. Stimulation of platelets in cats with severe HCM with the physiological agonist ADP resulted in increased P-selectin expression (mean fluorescence intensity) and soluble platelet-endothelial cell adhesion molecule-1 (sPECAM-1) compared to control cats. This study suggested that P-selectin and sPECAM expression may be useful biomarkers indicating increased platelet activation. Additionally, platelets from cats with HCM are procoagulant and these subjects maybe at a higher risk of ATE [57]. 

### 2.4. Urinalysis 

The association between CHF and renal disorder (cardio-renal axis) in dogs could be confirmed by urinalysis [16].

Renal concentrating ability is evaluated with urine specific gravity (USG), determined by refractometry. USG ranges from 1001 to 1080 in cats, and physiological values are higher than 1035. In hyposthenuria, the kidney retains some water balance functions, whereby solute is being reabsorbed in excess water and USG is less than 1008, being the ability to concentrate the urine lost in polyuric renal disease [58]. USG is usually measured for the assessment of renal concentrating ability when the animal is azotemic, polyuric, oliguric or anuric. Cats receiving diuretic therapy for heart failure will have a low urine specific gravity, and thus urine specific gravity should be evaluated before diuretic therapy is initiated [17]. 

Proteinuria must be interpreted at the same time of USG, and the Urinary Protein-Creatinine ratio (UPC) is a very useful tool to determine the greatness, and therefore the significance of proteinuria. Cardiac disease and extrarenal factors may cause a transitory mild prerenal proteinuria that increased glomerular permeability [58,59]. 

A novel marker of renal injury is CysC. It is a small protein that is reabsorbed and catabolized in the proximal tubules and therefore detectable urinary concentrations reflect tubulointerstitial damage. In a recent study, urinary CysC levels were reported under the border of detection in healthy cats, while in cats with CKD, the urinary CysC/urinary Cr ratio was significantly higher than in healthy subjects [24]. 

### 2.5. Effusion Analysis

Effusion is an abnormal accumulation of fluid within a body cavity (thoracic, pericardial, and abdominal) and it can be associated with several disorders in cats. Effusions are commonly classified in four groups according to their etiology and to cytological and biochemical criteria. There is transudate, which can be subtyped into protein poor and protein rich, exudate which can also subtyped in non-septic and septic, effusion caused by ruptured vessels or organs and effusion caused by cell exfoliation. Neoplastic effusion and reactive mesothelial hyperplasia are included in the last group. By analyzing these liquids, the ongoing pathological process could be better framed [60,61]. In a study of six cases with pericardial effusion, a population of immature lymphoid cells of medium-to-large size was observed with cytological diagnosis of lymphoma (three cases were classified as T-cell and three as B-cell lymphoma) [62]. CHF and portal venous hypertension can be associated with the formation of a protein-rich transudate, as it has been shown in 15.6% of 396 cats, as a result of increased intravascular hydraulic pressure in the liver or lung [61]. CHF is the most common cause of pericardial effusion and can be associated with HCM, unclassifiable cardiomyopathy, mitral valve disease, DCM, and pulmonic stenosis. Pericardial effusion and cardiac tamponade initially causes right-sided heart failure in cats [63]. Additionally, as reported by Hall et al., in 146 cases, cardiac disease (129) is the most common cause of pericardial effusion, and HCM was the most commonly diagnosed disease (39) followed by unclassified cardiomyopathy (31) and restrictive cardiomyopathy (19). Dilated cardiomyopathy (DCM) was less commonly found [28]. A retrospective study on 306 cats described CHF as the primary cause of pleural effusion, and it also suggested that a transudate, or a modified transudate, characterized by reduced cellularity, reduced protein level and low specific gravity, is indicative of underlying heart disease [29]. This data is confirmed by a recent study where CHF was the most common cause of pleural effusion, followed by neoplasia. Interestingly, patients in which effusion derived from CHF or neoplasia were significantly older than those with trauma or feline infectious peritonitis [64]. Cardiogenic chylothorax was described in some case reports associated with right-sided heart failure (derived from restrictive pericardial disease, heart-base chemodectoma, tetralogy of Fallot and tricuspid regurgitation, endocardial cushion defect and tricuspid dysplasia) and thrombosis of the cranial vena cava [65,66].

### 2.6. Vector Borne Disease

*Bartonella* spp. is a vector transmitted, Gram-negative bacteria that could be responsible of endocarditis in cats. The most common species in both humans and cats is *Bartonella henselae*, which is naturally conveyed between cats by the flea *Ctenocephalides felis felis*. Blood transfusion also represents a risk of transmission. Isolation of the bacterium is the gold standard, but because of the high prevalence of infection in healthy cats in endemic areas, a positive culture (or positive polymerase chain reaction) is not confirmed. Serology (IFAT or ELISA) is useful for exclusion of the infection because of the low positive predictive value compared to the good negative predictive value [67,68]. Rare case reports discuss *Bartonella henselae*-associated myocarditis or endocarditis. Fatal aortic and mitral valve *B. henselae*-associated endocarditis was reported in two cats in the USA [68,69]. In a case report, *B. henselae* was isolated from the blood of a cat using a novel culture approach that incorporates enrichment culture of patient samples in an optimized insect cell culture medium (Bartonella alpha Proteobacteria growth medium, BAPGM) [7]. Considering the difficulties in confirming *B. henselae* in cats, Palerme et al. recommended that positive blood results for the parasite could only be applied as major criteria when there is parallel documentation of resolution of clinical signs and echocardiographic changes in response to therapy [8]. 

Filarial worms are vector borne nematodes and *Dirofilaria immitis* is the most important species causing heartworm disease (HWD). The life cycle involves an intermediate mosquito host, but cats are imperfect hosts for dirofilarial worms. Pulmonary endo-mesoarteritis with occlusive medial hypertrophy are detected as immature worms arrive in the pulmonary vessels. The rare and short-term occurrence of microfilaremia and the low number of adult worms make it difficult to diagnose HWD in cats and a multistep approach, combining mainly antigen and antibody tests and diagnostic imaging is necessary [70,71,72].

Much rarer is *Hepatozoon silvestris* infection, associated in one cat to a severe myocarditis. In that case, clinical pathological signs revealed mild thrombocytopenia and slightly increased pancreatic lipase enzyme, while histopathology showed a severe histiocytic and lympho-plasmacytic myocarditis, and mature and developing protozoal meronts morphologically compatible with *Hepatozoon*. A polymerase chain reaction from the myocardium resulted in a 100% sequence identity with *H. Silvestri* [73].

### 2.7. Endocrinopathies

Hyperthyroidism is a common cardiac disease in cats [74]. In hyperthyroid cats, concomitant cardiac pathologies are very common, which could also be a direct consequence of the endocrinopathy [75]. Signs of cardiac disease, such as tachycardia, tachypnea, heart murmurs and sometimes even heart failure, are indeed often identified in hyperthyroid cats [76,77]. A recent study in cats evaluated the clinical-pathological characteristics and comorbidities present in cats with different degrees of hyperthyroidism [78]. It reported that most cats had a heart murmur and 45% showed evidence of cardiomegaly, diagnosed by ultrasound [78,79]. Interestingly, two cats included in the study and diagnosed with heart failure (1.4% of cases) were both severely hyperthyroid [78]. Previous studies reported a similar incidence of heart failure (2%–3%) in hyperthyroid cats [76,80]. The diagnosis of hyperthyroidism circulating thyroxine (T4) can be measured as a screening test, but for a definitive diagnosis, additional tests, such as free thyroxine (fT4), TSH (Thyroid stimulating hormone), T3 suppression test, TSH or TRH stimulation test, or the use of scintigraphy, may be necessary [74,75,81]. During hyperthyroidism in cats, there may be a slight increase in hematocrit, macrocytosis, the presence of Heinz bodies, an increase of ALT and ALP, and to a lesser extent, of blood urea nitrogen [75]. 

Cats with CHF caused by cardiomyopathies (i.e., hypertrophic cardiomyopathy) have increased plasma concentrations of tumor necrosis factor alpha (TNF-α). Heart failure is related to high left ventricle filling pressures, hypoxia and tissue ischemia, and stimulates monocyte activation with the release of cytokines (i.e., TNF-α and interleukin-6). Some research in feline with HCM have recognized insulin resistance and augmented growth hormone or insulin-like growth factor (IGF-1) concentrations [82,83,84]. A study on asymptomatic HCM cats revealed that 82% were hyperinsulinemic with insulin concentrations significantly above the laboratory reference range. Conversely, IGF-1 was not found to be significantly increased, even though some cats had high IGF-1 concentrations. Furthermore, no association was found between echocardiographic parameters, insulin or IGF-1 and cardiac biomarkers, leaving the role of insulin and IGF-1 uncertain in cats with HCM [13]. Cardiovascular abnormalities (e.g., left ventricular concentric hypertrophy, left atrial enlargement, and abnormal diastolic function) are often present in cats with acromegaly (hypersomatotropism) caused by functional somatotropic adenoma or hyperplasia of the pituitary gland. This leads to secondary increased concentrations of circulating insulin-like growth factor-1 (IGF-1), which can be measured by a commercially available radioimmunoassay [85,86].

## 3. Cardiac Biomarkers

Biomarkers are considered specific for the organ or tissue under study and they are released in proportion to the degree of injury and cardiac biomarkers, and therefore should provide information regarding diagnosis, prognosis or response to treatment. At present, in veterinary clinical practice, the two biomarkers with the highest predictability for cardiac diseases are: cardiac troponins I (cTnI) and N-terminal pro-brain natriuretic peptide (NT-pro BNP) [87,88]. 

### 3.1. Cardiac Troponin

Cardiac troponin is composed of three subunits (cTnT, C, and I) attached to the actin filament and plays a role in cardiomyocyte contraction. Injury to cardiomyocytes can result in troponins release and therefore an increase in their concentration may suggest a cardiac damage. Troponin concentration can also be a good quantitative measure of the extent of heart damage, but it provides no information on the cause of injury, as it cannot distinguish between primary cardiac and non-cardiac diseases with secondary heart involvement. The most sensitive and used troponin in veterinary medicine is troponin I [89,90]. In cats, various studies were performed comparing healthy subjects to patients with echocardiographic evidence of cardiac disease. These studies demonstrated significantly higher circulating troponin concentrations in cats with HCM and also in asymptomatic subjects [13,91,92,93,94,95]. Interestingly, an ultra-sensitive measurement method used in humans to measure cTnI concentration has been tested in cats showing to be applicable in these subjects [93,94,95]. In two of these studies, there was also a correlation of cTnI concentrations with the severity stage of heart disease [94,95]. In particular, Hertzsch et al. showed that cTnI concentration was able to discriminate, with a good sensitivity, even healthy cats from cats with asymptomatic heart disease. For this purpose, a cut-off value of <0,06 ng/mL was established, thus suggesting a possible application of this biomarker as a screening test [95]. In 2008, an increase in cTnI concentration was found in cats with kidney disease, as cardiac troponin is partly eliminated by renal excretion, thus confirming that kidney disease can result in their false increases. However, concomitant cardiac injury or cardiac injury resulting from renal disease cannot be excluded [96]. Other studies in cats also showed an increase in troponin concentration in cats suffering from hyperthyroidism and anemia [97,98,99] In conclusion, high levels of troponins are suggestive of myocarditis when other causes of severe myocardial injury have been excluded, considering, for example, that hypoxic stress and necrosis of cardiomyocytes are associated to cTnI release [100]. Additionally, cats with ATE had significantly higher median cTnI concentration compared to healthy cats or with mild or moderate HCM [95]. Some articles describe a transient myocardial thickening associated with high troponin values, suggestive of myocarditis. Troponins could be used to help in discriminating between hypertrophic cardiomyopathy and transient thickening caused by myocarditis, even if there is probably a clear overlap between the troponin values of the two categories [99,101]. Troponins have also been shown to be useful in evaluating the patient’s prognosis in cats [102,103]. 

In cats, various studies have been carried out with the aim of discriminating cats with dyspnea caused by cardiac pathology from cats with dyspnea caused by non-cardiac pathology by measuring cardiac troponin. These studies identified a significant difference between cardiac and non-cardiac causes, however showing a considerable overlap of the values of the two groups of cats, thus suggesting a poor clinical utility of this biomarker for the identification of cardiac dyspnea in cats [104,105,106,107]. 

### 3.2. Natriuretic Peptides

Natriuretic peptides are a group of structurally related proteins that mainly include the atrial natriuretic peptide (ANP), the brain natriuretic peptide (BNP) and the C-type natriuretic peptide. ANP is released from atrial myocardium, while BNP is released from both atrial and ventricular myocardium, but in a higher concentration in the latter, and are then cleaved into their inactive forms [*amino-terminal atrial natriuretic peptide* (NT-pro-ANP) and *amino terminal pro-brain natriuretic peptide* (NT-pro-BNP)] and their active forms [*C terminal atrial natriuretic peptide* (CANP) and *C-terminal brain natriuretic peptide* (CBNP)]. The most studied natriuretic peptide in veterinary medicine is the N-terminal fragment of BNP (NT-pro-BNP), also because this component is characterized by a longer half-life. Natriuretic peptides are released following a myocardial stretch (increased preload) and play a role in cardiovascular and cardiorenal homeostasis; they antagonize the effects of the renin-angiotensin-aldosterone system (RAAS) (hyperactive during heart disease) by mainly promoting natriuresis, diuresis and vasodilation [108]. Various studies have been carried out to compare natriuretic peptides concentrations in healthy cats and in cats with cardiac disease diagnosed by echocardiography [109,110,111,112,113,114,115,116,117,118,119,120]. NT-pro-BNP was reported to be significantly higher in cats with heart disease in all studies, and some of them identified an optimal NT-pro-BNP cut-off value for the identification of occult cardiomyopathy with high sensitivity and specificity, using both traditional quantitative ELISA methods (first and second generation) and SNAP ELISA methods: between 100 and 122 pmol/L [109,110,111,112,115,117,118,119]. Echocardiography remains preferable for the screening of mild or early-stage diseases, as the ability of NT-pro-BNP to identify mild degrees of pathology proved to be less accurate [109,111,112,117,119]. ANP and NT-pro-ANP also showed significant increases in cats with heart disease in some studies, but showed less sensitivity and less accuracy in identifying cats with occult cardiomyopathy [114,116,119,121].

Natriuretic peptides are also used to differentiate dyspnea caused by cardiac causes from dyspnea caused by non-cardiac causes, which are especially useful in emergency situations where other collateral tests are not available [122]. A higher average NT-pro-BNP value in cats with dyspnea and with pleural effusion of cardiogenic origin, also showing the possibility of measuring this biomarker both on plasma and pleural liquid, through quantitative first- and second-generation ELISA methods and rapid SNAP ELISA methods has been shown. In this last case, it has been necessary to dilute the pleural effusion with a saline solution to increase its specificity [115,123,124,125,126,127].

Recently, the measurement of NT-pro-BNP in combination with thoracic radiography demonstrated an improvement in diagnostic accuracy in determining heart failure in cats presented with dyspnea, compared to thoracic radiography alone [128].

Like troponins, natriuretic peptides may increase in non-cardiac diseases secondarily affecting the heart. NT-pro-BNP and NT-pro-ANP were found elevated in cases of hyperthyroidism and kidney disease with severe azotemia [98,129,130,131].

Finally, NT-pro-BNP and NT-pro-ANP were suggested as survival times predictors and therefore as prognostic values [104,116].

## 4. Conclusions

This manuscript describes the available data on cardiac clinical pathology and analyzes all the aspects related to their practical use, subdividing them into classical analytes and biomarkers. Such a review may be useful for the clinician, the clinical pathologist, and the researcher as it furnishes hints on the use of cardiac markers both directly applicable and to be further investigated.

## Data Availability

Not Applicable.

## Abbreviations

Activated partial thromboplastin time (aPTT)

Acute-phase proteins (APPs)

Brain natriuretic peptide (BNP)

Alanine aminotransferase (ALT).

Alkaline phosphatase (ALP)

Arterial thromboembolism (ATE)

Aspartate aminotransferase (AST)

Atrial natriuretic peptide (ANP)

Cardiac troponins I (cTnI)

Cardio renal syndrome (CRS)

Cardiovascular-renal disorders (CvRD)

Catalase (CAT)

Cystatin C (CysC)

Creatinina (Cr)

Creatine kinase (CK)

Chronic kidney disease (CKD)

Congestive Heart Failure (CHF)

Diabetes mellitus (DM)

Dilated cardiomyopathy (DCM),

Fibrinogen/fibrin degradation product (FDP)

Glomerular filtration rate (GFR)

Glutathione peroxidase (GPx)

Heartworm disease (HWD)

Heart failure (HF)

Hypertrophic cardiomyopathy (HCM)

Insulin-like growth factor (IGF-1)

Lactate dehydrogenase (LDH)

N-terminal pro-brain natriuretic peptide (NT-pro BNP)

Plasma thrombin-antithrombin (TAT)

Prothrombin time (PT)

Reactive oxygen species (ROS)

Red blood cell distribution width (RDW)

Serum amyloid A (SAA)

Symmetric dimethylarginine (SDMA)

Superoxide dismutase (SOD)

Tumor necrosis factor alpha (TNF-α)

Urea (Ur)

Urinary Protein-Creatinine ratio (UPC)

Urine specific gravity (USG)
